# Relationship Between Handgrip Strength and Framingham Risk Score in Older Adults in Bushehr, South Iran

**DOI:** 10.1155/jare/7101568

**Published:** 2026-11-09

**Authors:** Anahita Ghasemi, Morteza Azari, Maryam Marzban, Mohammadreza Kalantarhormozi, Sadegh Darvishi

**Affiliations:** ^1^ Department of Internal Medicine Faculty of Medicine Bushehr University of Medical Sciences Bushehr Iran bpums.ac.ir; ^2^ Cardiovascular Research Center Shiraz University of Medical Sciences Shiraz Iran sums.ac.ir; ^3^ QIMR Berghofer Medical Research Institute Royal Brisbane Hospital Brisbane Queensland Australia qimrberghofer.edu.au

**Keywords:** aged, cardiovascular diseases, Framingham Risk Score, geriatrics, hand strength

## Abstract

**Background:**

Handgrip strength (HGS) indicates neuromuscular function, important in the elderly. Previous studies showed that HGS can be used as a predictor of cardiovascular disease (CVD)–related death. This study aimed to identify the relationship between HGS and the Framingham Risk Score (FRS) in the elderly referring to Bushehr Hospital in 2019.

**Methods:**

In the Bushehr Elderly Health (BEH) Program, 3000 individuals aged ≥ 60 years who had lived in Bushehr for at least 1 year, had no plans to relocate within the following 2 years, and had stable physical and mental conditions were selected using a cluster sampling method. Of these, 2426 participants completed the second phase of the study. Demographics and FRS‐related information were recorded; CVD risk was assessed as low, medium, or high based on this score. Total, relative, dominant, and nondominant HGSs were measured using a DHD‐1 dynamometer device. Association of HGS with FRS was evaluated by regression analysis, performed with STATA MP 15.

**Results:**

Of 2426 participants, 48.5% were men; majority were at high risk for CVD. Mean HGS differed significantly across the groups. It increased from low (17.21 ± 5.22) to medium (18.33 ± 7.66) and high (24.70 ± 9.45, *p* < 0.001). Unadjusted analysis showed a weak positive association for mean HGS, dominant HGS, and nondominant HGS (*β* ≈ 0.3). Relative HGS was strongly associated with FRS (*β* = 8.6). After adjustment, all HGS types lost their significance.

**Conclusions:**

HGS and FRS were not associated in adjusted analysis of this study. This indicates a stronger role for demographic and clinical variables rather than absolute, relative, dominant, or nondominant HGS. Given the limitations of this study, further research is required before drawing definitive conclusions.

## 1. Introduction

As humans age, muscle strength, mass, and quality decrease. This decline results in functional impairment and weakness in the elderly [1]. Measuring the force of squeezing an object as tightly as possible with the hands using a handheld dynamometer, known as handgrip strength (HGS), has been suggested as an easy, noninvasive method for evaluating muscle strength without specialized tools or high costs [2]. Total HGS, as well as dominant‐ and nondominant‐hand HGS, are suggested to be measured for distinguishing laterality (possible asymmetry) and investigating neuromuscular function more comprehensively [3, 4]. Furthermore, it has been suggested for identifying frailty among patients with vascular diseases [5] and serves as a predictor of chronic diseases and all‐cause mortality in middle‐aged and older people [6]. Studies on the general population have also suggested HGS as a good predictor of cardiovascular disease (CVD) mortality [7], similar to systolic blood pressure (SBP) [8, 9]. Also, HGS has been associated with the incidence of heart disease among Europeans [10, 11] and with metabolic syndrome (MetS) in other populations, such as Korea [12].

In middle‐aged adults and older adults, relative HGS, which considers HGS of both hands, normalized based on body mass index (BMI), has been shown to have distinct clinical value, being associated with CVD biomarkers, including SBP, serum triglycerides (TG), insulin, glucose, and serum levels of high‐density lipoprotein cholesterol (HDL‐C) [13, 14]. Some studies have reported that relative HGS is associated with a greater number of variables compared with absolute HGS in young healthy populations [15]. Although the most accurate measure for the geriatric population has yet to be investigated, the available literature suggests that both absolute and relative HGS should be measured in primary care for CVD risk assessment.

The Framingham Risk Score (FRS) is a simple and commonly‐used tool for assessing the risk of developing CVD over a 10‐year period [16]. It has been found to be associated with motor function (walking speed, chair rises, balance, finger tapping, and grip strength) in early midlife, independent of its effect on cognitive impairment and dementia [17]. Yet, its association with HGS remains controversial, as some studies have reported a negative association between FRS and HGS [18, 19], while other researchers have suggested a significant association only in men, with no association between dominant HGS and FRS in either sex [14]. Therefore, it is not yet clear which handgrip measurement can be used in public health and clinical practice for assessing CVD risk and mortality.

In Iran, few studies have examined the relationship between HGS and CVD risk factors, mainly focusing on adults [20], while older adults appear to have been largely overlooked in this area of research. Given the need for simple and low‐cost screening methods in primary care, establishing the potential role of HGS in risk assessment is particularly relevant. However, only one study has been performed in the Iranian population aged 35–65 years, which reported no association between relative HGS and lipid profile measures, including serum levels of total cholesterol (TC), TG, HDL‐C, and low‐density lipoprotein cholesterol (LDL‐C) [20]. Considering the lack of sufficient studies in the Iranian population, especially in the elderly population, and the controversial results of limited investigations, in this study, we examined the association of HGS with FRS in the elderly population. Considering the significance of laterality in HGS, and the difference between laterality and normalization methods (HGS/BMI) [21], we considered all types of HGS, including relative, dominant, and nondominant HGS, separately.

## 2. Materials and Methods

### 2.1. Study Design and Participants

This study is an observational analytical study, conducted using data from the Bushehr Elderly Health (BEH) Program, a population‐based prospective cohort study of community‐dwelling older adults in Bushehr, southern Iran. The first phase was conducted in 2013–2014 using cluster sampling on all elderly individuals (aged ≥ 60 years) who had lived in Bushehr for at least 1 year before the start of the study, had no plans to leave Bushehr for the next 2 years after enrollment, had appropriate physical and mental abilities, and had no underlying diseases, such as stroke, vascular, or neuromuscular disorders, with approximately 91% population coverage and 3000 participants enrolled in Phase I. Written informed consent was obtained from all participants prior to enrollment in the BEH program.

For participant selection, multistage random sampling was carried out based on the six classifications of the Bushehr Provincial Management and Planning Organization. In total, 75 classes were defined, and the sample size of each class was determined according to the number of households living in that class. Each block consisted of several clusters, defined as groups of houses separated from other houses by alleys. A total of 78 blocks were created. Three blocks were excluded because they were located in military air and naval bases and in the Halila area, which was situated on the outskirts of the city. This resulted in 75 blocks being used as the sampling areas. The eligible participants were selected based on the abovementioned inclusion criteria, resulting in a total of 3000 participants in Phase I. Data from participants in the Phase I BEH cohort were used to report the demographic and baseline clinical characteristics of the general study population.

The first follow‐up (Phase II) was conducted in 2015–2017 to evaluate repeated clinical assessments. Of the Phase I cohort, 2772 participants were eligible for Phase II. Following dropouts due to death, migration, or lack of consent to continue the study, 2426 participants completed the second phase (Figure 1). Data from these participants (*n* = 2426) were used for the analytical phase of the study, including HGS measurements and univariate regression analysis. No randomization or sample size calculation was applied for this analytical phase.

**FIGURE 1:**
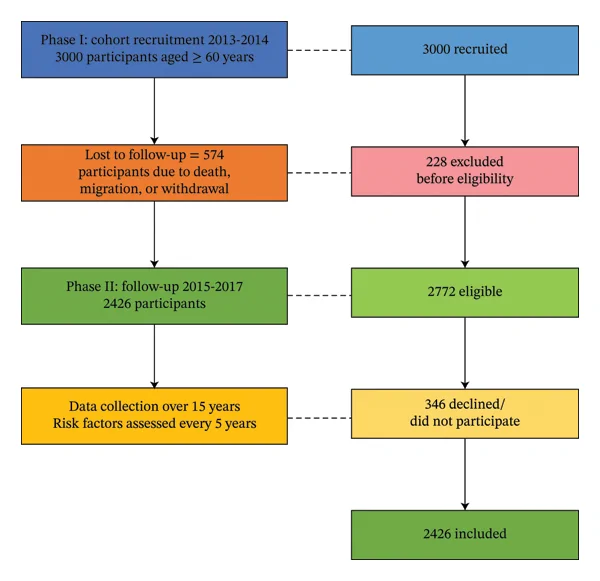
Participant flow diagram illustrating the inclusion and exclusion of participants in the observational cohort study, from the Phase I cohort to the Phase II follow‐up.

### 2.2. Hand Strength Measurement

HGS measurement was performed using a DHD‐1 dynamometer according to standardized protocols, after calibration to ensure measurement accuracy. The handle was placed with the clamp positioned below, and the individual’s hand was secured on the clamp with a safety strap. The participant was seated straight and aligned with the device. The shoulder was extended to the side, and the elbow was placed at a 90‐degree angle. The hands rested in a neutral and comfortable position. The device was applied smoothly, without rapid, jerky, or painful movements. The wrist angle was observed to be between 0 and 30 degrees. If it exceeded 30 degrees, it was recorded. The measurement was repeated for both hands of each participant, and the dominant hand was determined based on self‐reported hand preference. Each test was repeated three times, with a short rest between each attempt (when the participant felt ready for the next experiment) to determine the participant’s maximum effort, recorded as the final HGS of that hand, and confirmed only if the values were within a 10% difference.

After accurate measurement, we reported two measures separately to distinguish laterality from normalization approaches:

a. The HGS normalization measures, including absolute (total) HGS, and HGS of each hand (dominant and nondominant).
b. Relative HGS, calculated as the sum of dominant and nondominant HGS divided by BMI.

The healthcare personnel who performed the measurements were trained by a professor of internal medicine, who observed and validated their performance and certified them as eligible. These personnel were not involved in other phases of the study and performed only the HGS measurements. To ensure consistency across measurements, quality control was maintained through device calibration, standardized measurement procedures, repeated measurements, and confirmation of measurements within a predefined 10% range.

### 2.3. Blood Sample

A total of 24 mL venous blood samples were taken after 8–12 h of fasting. The samples were collected in EDTA‐containing tubes (2 mL), heparinized tubes (3 mL), and the remainder in plain tubes with a clotting activator for the extraction of serum, plasma, cell sediments, and whole blood. EDTA‐containing tubes were used for the measurement of hemoglobin (Hb)‐A1c and complete blood count (CBC); serum was separated from the heparinized tubes by centrifugation and divided into four tubes. Two microtubes were used for this study, and the rest were stored. The whole blood sample was used for the measurement of fasting blood sugar and lipid profile. All tests were performed in the Bushehr laboratory. The presence of diabetes mellitus (DM) was assessed by serum testing (defined by fasting blood sugar ≥ 126 mg/dL, HbA1c ≥ 6.5%, oral glucose tolerance test ≥ 200 mg/dL, or random plasma glucose ≥ 200 mg/dL, in the presence of clinical symptoms) [22] and by self‐report from participants who reported a previous diagnosis of DM by a physician.

### 2.4. The FRS

The FRS was calculated based on age, sex, TC, HDL‐C, smoking habits, DM, and SBP. Participants were categorized into three risk groups: low risk (< 10%), medium risk (10%–20%), and high risk (> 20%). In men, these corresponded to total scores of 3–10, 11–14, and ≥ 15, respectively. In women, the categories were defined as 3–12, 13–17, and ≥ 18, respectively [23].

For calculating FRS and other objectives of the study, a complete history was taken from the participants, recording demographics (including age, sex, marital, and socioeconomic status). In addition, a complete physical examination was performed, which included weight and height, used for calculation of BMI, waist circumference (WC), and hip circumference (HC), as well as systolic and diastolic blood pressure, with blood tests. To measure height, the subject was asked to stand with the head, shoulders, hips, knees, and ankles perpendicular to the measuring rod. The outer ear orifice was positioned along the lower edge of the eye socket. A horizontal ruler was placed on the forehead and gently moved to the top of the person’s head, pressing the hair to measure height, recorded in cm. For measuring participant’s weight, a Secca digital scale was used. BMI was calculated using the formula weight/height^2^. Blood pressure was measured by an arm digital scale (Emeron, made in Germany) after 15 min of resting. It was measured twice with an interval of 10 days, and the mean of the two measurements was considered as the final value.

### 2.5. Statistical Analysis

Quantitative variables were described using mean and standard deviation (SD), after confirming the normal distribution of variables by Kolmogorov–Smirnov test. Categorical data were presented by frequency and percentage. First, we categorized the participants into low‐, medium‐, and high‐risk groups, based on FRS categories, and compared the variables among the subgroups. Then, we calculated the adjusted and unadjusted association of the three HGS measurements with total FRS to identify the risk factors, reporting beta coefficient, standard error (SE), and 95% confidence interval (CI). STATA MP 15 was used to analyze the data. Regression models were adjusted for demographic and clinical variables that have been found to be associated with HGS (including age, sex, BMI, lipid profile, blood pressure, and fasting blood sugar) [24], though some may overlap with factors included in FRS, and model assumptions were checked. The assumptions of linear regression, including linearity, independence, homoscedasticity, and approximate normality of residuals, were assessed before interpreting the regression models. A *p* value less than 0.05 was considered statistically significant.

### 2.6. Ethical Considerations

This study was a part of the Bushehr Health Cohort Study, funded by Bushehr University of Medical Sciences and the Institute of Metabolism and Endocrine Research. It was approved and registered by the Ministry of Health with the endorsement of the Higher Education Institutions of Iran on March 11, 2017, under the code IR.BPUMS.REC.1400.197. Ethical considerations were observed throughout the study in accordance with the guidelines of the Declaration of Helsinki. These included obtaining the necessary permissions from relevant authorities, assuring confidentiality of information, ensuring the freedom of participants to join or withdraw from the study, and maintaining scientific integrity at all stages.

## 3. Results

The demographics and baseline characteristics of the 3000 participants, representing the general population of the Phase I cohort, are shown in Table 1. As depicted, participants were categorized into three risk groups of low‐, medium‐, and high‐risk, according to their FRS; of these, 227 participants were in the low‐risk group, 700 in the medium‐risk group, and 2073 in the high‐risk group. The demographic and baseline characteristics of the participants, including mean age, distribution of sex, marital status, BMI, WC, SBP, HbA1c, TC, HDL and LDL, and total FRS, were compared among the three risk groups in the general population of the Phase I cohort (*n* = 3000).

**TABLE 1:** The demographic and clinical characteristics of the three groups, based on Framingham Risk Score categories.

Variable	Categories	Total (*N* = 3000)	Low risk (*N* = 227)	Medium risk (*N* = 700)	High risk (*N* = 2073)	Value	*p* value
Age (years), mean ± SD		69.34 ± 6.39	66.83 ± 5.24	68.45 ± 6.12	70.13 ± 6.53	39.87^∗^	< 0.001^‡^

Sex	Male	1455 (48.5)	2 (0.88)	83 (11.86)	1370 (66.09)	839.10^†^	< 0.001^§^
	Female	1545 (51.5)	225 (99.12)	617 (88.14)	703 (33.91)		

Marital status	Single	25 (0.83)	0	13 (1.86)	12 (0.58)	63.98^†^	< 0.001^§^
	Married	2248 (74.93)	163 (71.81)	453 (64.71)	1632 (78.73)		
	Divorced	26 (0.87)	3 (1.32)	10 (1.43)	13 (0.63)		
	Widowed	701 (23.37)	61 (26.87)	224 (32)	416 (20.07)		

BMI (kg/m^2^), mean ± SD		27.51 ± 4.90	28.26 ± 5.09	28.41 ± 5.23	26.98 ± 4.63	26.94^∗^	< 0.001^‡^

WC (cm), mean ± SD		98.71 ± 12.01	97.39 ± 11.68	99.65 ± 12.86	98.47 ± 11.62	3.96^∗^	0.0223^‡^

SBP (mmHg), mean ± SD		139.65 ± 19.32	123.3 ± 12.19	136.82 ± 17.58	143.46 ± 19.46	137.29^∗^	< 0.001^‡^

TC (mg/dL), mean ± SD		182.18 ± 44.12	197.60 ± 4.41	189.73 ± 46.33	176.31 ± 42.54	46.06^∗^	< 0.001^‡^

LDL‐C (mg/dL), mean ± SD		19.43 ± 37.70	119.81 ± 36.53	113.85 ± 40.91	105.78 ± 35.77	23.36^∗^	< 0.001^‡^

HDL‐C (mg/dL), mean ± SD		45.94 ± 11.21	54.5 ± 11.3	49.79 ± 11.9	42.83 ± 9.9	208.16^∗^	< 0.001^‡^

HbA1c (mg/dL), mean ± SD		5.67 ± 1.56	4.99 ± 0.76	5.48 ± 1.43	5.86 ± 1.66	41.49^∗^	< 0.001^‡^

FRS, mean ± SD		23.78 ± 8.10	7.06 ± 1.53	14.39 ± 3.00	28.78 ± 2.53	13,270.50^∗^	< 0.001^‡^

Abbreviations: BMI, body mass index; FRS, Framingham Risk Score; HbA1c; hemoglobin A1c; HDL; high‐density lipoprotein cholesterol; LDL‐C, low‐density lipoprotein cholesterol; TC, total cholesterol; WC, waist circumference.

^∗^The *F* value.

^†^The *X* ^2^ value.

^‡^The results of one‐way ANOVA test.

^§^The results of chi‐square test. Data in this table are presented for 3000 participants (Phase I of the cohort).

Among the 2426 participants who completed Phase II, mean total HGS was 22.16 ± 9.23 and mean relative HGS was 0.83 ± 0.38. As shown in Table 2, mean total and relative HGS values differed significantly between the three risk groups, with higher mean values observed in higher‐risk groups (*p* < 0.001).

**TABLE 2:** The mean hand strength measurements of the three groups, based on Framingham Risk Score categories.

Variable	Categories	Total (*N* = 2426)	Low risk (*N* = 225)	Medium risk (*N* = 702)	High risk (*N* = 1499)	*p* value
Hand strength, mean ± SD	22.16 ± 9.23	17.21 ± 5.22	18.33 ± 7.66	24.70 ± 9.45	183.87^∗^	< 0.001^†^
Relative hand strength, mean ± SD	0.83 ± 0.38	0.62 ± 0.21	0.66 ± 0.32	0.94 ± 0.39	204.24^∗^	< 0.001^†^

^∗^The *F* value.

^†^The results of one‐way ANOVA test. Data in this table are presented for 2426 participants (Phase II of the cohort).

The results of the unadjusted univariate regression analysis showed a weak positive association of total, dominant, and nondominant HGS with FRS, whereas relative HGS showed a stronger association (*β* = 8.6; Table 3). After adjustment for demographic and clinical variables (including age, sex, BMI, lipid profile, blood pressure, and fasting blood sugar), none of the HGS measures remained significantly associated with FRS (*p* > 0.05; Table 3).

**TABLE 3:** The association of variables with Framingham Risk Score based on unadjusted and adjusted univariate linear regression.

Variable		Beta	95% confidence interval		Standard error	*p* value
			Lower limit	Upper limit		
Total hand strength	Unadjusted	0.350	0.316	0.382	0.017	< 0.001
	Adjusted	−0.004	−0.381	0.288	0.017	0.785

Relative hand strength	Unadjusted	8.571	7.769	9.372	0.408	< 0.001
	Adjusted	0.185	−0.678	1.050	0.440	0.674

Dominant hand strength	Unadjusted	0.332	0.300	0.365	0.016	< 0.001
	Adjusted	−0.005	−0.036	0.025	0.015	0.713
	Adjusted	−0.001	−0.034	0.304	0.016	0.913

*Note:* Data in this table were calculated using data from 2426 participants (Phase II of the cohort).

## 4. Discussion

In this study, we pursued two objectives; the main objective was to analyze the association between FRS and HGS, considering the significant value of FRS for estimating CVD risk. The results showed that the mean total and relative HGS differ among the three risk groups, indicating a significant effect of CVD risk on HGS. However, the significant association observed in the unadjusted analyses was attenuated after adjustment for conventional CVD risk factors. This finding suggests that the predictive contribution of HGS to FRS may be largely explained by established CVD risk factors incorporated into the FRS calculation. Therefore, while HGS was not independently associated with FRS in the fully adjusted models of this study, these findings should not be interpreted as evidence that HGS lacks clinical value in CVD risk assessment.

These results are similar to those reported in Taiwan, which suggested that the relative HGS was only associated with FRS in men, while dominant HGS was not associated with FRS in either sex [14]. Although that study was nationwide, the mean age of their study population (65.6 ± 9.4 years) was not far from that of the present study. A published abstract focusing on a randomly‐selected, community‐dwelling cohort of middle‐aged to elderly men residing in Adelaide, Australia, also showed that mean HGS did not have a prognostic value for CVD risk assessment beyond standard FRS factors [25]. These results are consistent with those of the present study, though the age and race of the selected populations differed. Some have suggested relative HGS as a more accurate tool, as it includes calculation of HGS from both hands and incorporates BMI in its calculation. Therefore, we evaluated it separately; however, the results of multivariate regression were nonsignificant, and the controversy continues regarding this index, as well as other HGS indices. Future studies should directly compare the diagnostic and predictive performance of absolute, relative, dominant, and nondominant HGS measures across different age and sex groups to determine which measure, if any, provides the most useful information for CVD risk assessment in older adults.

There is unfortunately limited evidence available regarding the association of HGS and FRS, though some studies have addressed the association of HGS with other CVD biomarkers, yielding controversial results. In an Iranian study on a population aged 35–65 years, relative HGS was found not to be associated with serum levels of TC, TG, HDL‐C, and LDL‐C [20]. These results are consistent with this study, considering the evaluation of an Iranian population and the nonsignificant findings obtained; however, the age of the studied population and the CVD biomarkers evaluated differed from those of the present study. In contrast, other studies have shown significant associations between relative HGS and HDL‐C, as well as MetS in both sexes [14]; between HGS and other CVD biomarkers, including SBP, TG, insulin, glucose, and HDL‐C [13] in the general population; and between HGS and the incidence of heart disease [10, 11] and MetS [12] in the elderly. As we did not analyze the components of FRS separately with HGS, we cannot comment on this based on the results presented. Nevertheless, the controversy regarding CVD biomarkers continues. For example, another study on a population aged 50–75 years showed significant associations between HGS and SBP in both sexes, LDL‐C and DBP in men, and TG and uric acid in women. However, after multivariate analysis, these variables lost their significance, and only WC, fat mass, and high‐sensitivity C‐reactive protein (hs‐CRP) remained significant in both sexes, while BMI and leptin remained significant in men [26]. The varied characteristics of populations (including age and sex distribution) and variables studied may underlie the differences observed. Therefore, further studies are required to determine the role of HGS in predicting CVD biomarkers, and especially FRS, in the elderly.

Other studies evaluating FRS have not focused on HGS, but on other similar variables. In one study emphasizing the value of FRS in the elderly for predicting future frailty, it was shown that its effect disappeared after adjusting for age. In that study, HGS was evaluated as part of weakness, along with slowness, exhaustion, low activity level, and weight loss—three of which were considered indicators of frailty [27]. This study was similar to the present one in terms of nonsignificant results obtained in the elderly population, though the measured variable differed, as we did not directly measure frailty factors or sarcopenia in the participants. In another study on very old individuals, FRS was reported not to be associated with CVD mortality in this specific population, while only homocysteine showed an association [28]. Other studies have also referred to increases in FRS in the elderly related to homocysteine, uric acid, ferritin, creatinine, and TG levels [29]. Differences in these biomarkers across populations may underlie the diverse results observed in the assessment of FRS.

The second objective of this study was the overall assessment of FRS, categorized into low‐, medium‐, and high‐risk groups for CVD assessment in the cohort population. The results showed that 94.1% of elderly men and 45.4% of elderly women were in the high‐risk group. These statistics are considerably high and indicate the need for further screening and diagnostic measures in this population. In another study on the Iranian population (Northern Iran), 49% of men and 12% of women were classified as high‐risk for CVD based on FRS [30]. Therefore, it is necessary to include FRS and its parameters in the primary care of the elderly in Bushehr, particularly men.

### 4.1. Strengths and Limitations

This study had several strengths. First, we performed this study on a large population as part of a cohort study and examined several variables simultaneously, which cannot be achieved in small cross‐sectional studies. Moreover, cohort studies allow researchers to examine temporal relationships between variables and assess whether exposure to a potential risk factor precedes the development of an outcome, though they cannot establish causality on their own. Another point to be acknowledged is that we reported both laterality measures (dominant and nondominant HGS) and their normalization by BMI (relative HGS), which is one of the strengths of this study, as most previous studies have measured either dominant or nondominant HGS (without considering functional asymmetry) or have not considered normalization measures. However, we did not evaluate the diagnostic accuracy of each measure in this study to identify which is clinically more accurate. Therefore, to obtain more reliable results, validated measurement instruments with established reliability and validity are needed to accurately assess the variables of interest. Also, there are other limitations that should be acknowledged. First, evaluating a large population can lead to considerable diversity in the study population, such as age heterogeneity, sex distribution, educational level, income status, and psychological status, which could have influenced the results but were not included in this study. Thus, the reported associations may vary when different subgroups are considered separately; however, in this study, we only aimed to report the overall associations. Furthermore, considering the observational nature of the study, we did not report detailed diagnostic measures such as variance inflation factors, which may limit a full assessment of multicollinearity. Moreover, we adjusted the associations for variables also included in FRS to explore whether HGS provides information beyond conventional risk factors, which reduces the ability to detect independent associations, and caution should be taken in the general interpretation. Furthermore, we only evaluated FRS and its parameters, which, though valuable, cannot capture all dimensions of heart disease. Also, the high proportion of participants falling into the high‐risk group may have caused a potential ceiling effect for FRS in this population, hindering its ability to detect associations with HGS.

## 5. Conclusions

From a clinical perspective, our findings do not support the use of HGS as an independent screening tool for estimating 10‐year CVD risk in the elderly. Nevertheless, this conclusion should be interpreted with caution, as factors such as sample characteristics (including age heterogeneity and sex distribution), population structure, and unmeasured confounders may have influenced the results. Therefore, additional studies are required to investigate the associations in different subgroups before dismissing HGS entirely. It is also important to compare the accuracy of the available metrics, using appropriate validation methods and clearly defined performance criteria, to improve clinical practice in the geriatric population. This was beyond the objectives of the present study and is recommended for future investigation.

**Nomenclature**
HGS: Handgrip strength
CVD: Cardiovascular disease
SBP: Systolic blood pressure
TG: Triglycerides
HDL‐C: High‐density lipoprotein cholesterol
FRS: Framingham Risk Score
BMI: Body mass index
TC: Total cholesterol
LDL‐C: Low‐density lipoprotein cholesterol
BEH: Bushehr Elderly Health
DM: Diabetes mellitus
WC: Waist circumference
HC: Hip circumference
CBC: Complete blood count
HbA1c: Hemoglobin A1c
SD: Standard deviation
SE: Standard error
CI: Confidence interval
MetS: Metabolic syndrome
hs‐CRP: High‐sensitivity C‐reactive protein

## Author Contributions

Anahita Ghasemi: data curation and writing–original draft

Morteza Azari: data curation and writing–review and editing.

Maryam Marzban: formal analysis, software, and writing–original draft.

Mohammadreza Kalantarhormozi: conceptualization, supervision, and writing–review and editing.

Sadegh Darvishi: formal analysis, software, and writing–review and editing.

## Funding

This research did not receive any specific grant from funding agencies in the public, commercial, or not‐for‐profit sectors.

## Disclosure

All authors read and approved the final version of the manuscript.

## Conflicts of Interest

The authors declare no conflicts of interest.

## Data Availability

The data that support the findings of this study are available from the corresponding author upon reasonable request.
